# Clear Cell Sarcoma of the Foot in an 18-Year-Old Female

**DOI:** 10.1155/2019/8378106

**Published:** 2019-11-23

**Authors:** Marwan A. Albeshri, A. Ashour

**Affiliations:** College of Medicine, King Abdulaziz University, Jeddah, Saudi Arabia

## Abstract

We report a case of an 18-year-old female without a relevant medical history who presented with an 8-month history of a left foot mass. It started as a small nodule that progressively increased in size over time. The mass then became ulcerative with foul-smelling discharge. There was no palpable left inguinal or other lymph nodes upon physical examination. Histological examination of the biopsy confirmed a diagnosis of clear cell sarcoma. Clear cell sarcoma is a rare soft tissue neoplasm. However, early diagnosis is crucial to prevent metastasis and worsened prognosis. Clear cell sarcoma has an extremely poor prognosis once metastasis occurs, and to the best of our knowledge, only fewer than 100 cases have been reported in the literature.

## 1. Introduction

Clear cell sarcoma (CCS) is a rare soft tissue tumor which involves tendons or an aponeurosis in almost all cases. It is estimated that CCS represents only one percent of all sarcomas. Nevertheless, an actual incidence rate has not yet been established [1, 2].

There are multiple challenges when diagnosing CCS. First, the rare nature of the neoplasm may delay its diagnosis and prevent early intervention to prevent metastasis.

Second, CCS shares common histological and immunohistochemical features with malignant melanoma (MM), which might make the differentiation between the two neoplasms more difficult. CCS is characterized histologically by uniform polygonal to fusiform eosinophilic or clear cells with central round nuclei and prominent basophilic nucleoli [3–8]. CCS can be differentiated from MM by molecular biology testing, such as RT-PCR and FISH, and by characteristic chromosomal abnormalities and the genetic translocation t(12;22)(q13;q12). CCS can also be differentiated clinically by extension of the lesion within the cutaneous layer and the susceptibility to lymph node and lung metastasis [9, 10].

CCS most commonly affects young Caucasian adults with a 1 : 1 male to female ratio [5]. CCS commonly presents as a slow-growing, painless mass that most frequently affects the lower limbs, particularly around the ankle region. However, more advanced lesions may present with other systemic symptoms, such as weight loss, anorexia, and fatigue. The five-year survival rate is sixty-three percent, and the tumor size is considered one of the prognostic factors in patients with CCS [2–7].

In this manuscript, we report a case of CCS affecting the left foot of an 18-year-old Saudi female that presented as an ulcerating mass with foul-smelling discharge.

## 2. Case Presentation

**Figure 1:**
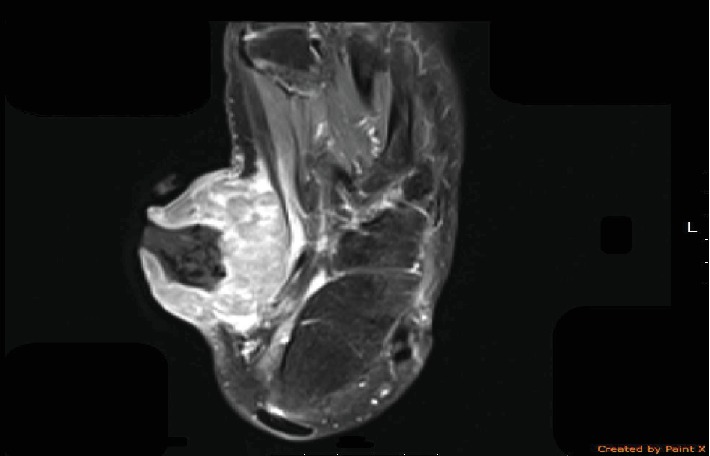
MRI of the left foot showing a large exophytic soft tissue mass in the medial side with necrotic center. The lesion involves the flexor hallucis longus and the abductor hallucis muscles.

An 18-year-old female without a relevant medical history presented with an 8-month history of a left foot mass. It started as a small nodule that progressively increased in size over time, and another small mass developed beside it two weeks before the diagnosis. The patient started to develop fever, rigor, and chills. She underwent incision and drainage, and antibiotics were prescribed for her in a primary health care center, which delayed the diagnosis for two more months. On examination, there was a 6×6 cm rounded, firm mass with a mass of approximately 1.5 cm extending from it. The large mass had an open wound with foul-smelling, purulent discharge leaking from the wound with no other masses on the same side. There were no palpable left inguinal or other lymph nodes upon physical examination. Magnetic resonance imaging (MRI) of the left foot showed a large exophytic, lobulated, and enhancing mass, measuring 6 × 4.5 × 6 cm with central necrosis in the soft tissue of the midfoot. The lesion also involved the flexor hallucis and abductor hallucis muscles (Figure 1). Six core punch biopsies were taken from the large and small masses and confirmed a diagnosis of CCS. Computed tomography (CT) of the abdomen and pelvis was also performed, which revealed multiple enlarged lymph nodes in the left inguinal region and on the left side of the abdominal aortic bifurcation. Lung and abdominal CT were performed and showed no signs of distant metastasis. The patient underwent a left below-the-knee amputation as a palliative procedure. Pathological examination of the amputated specimen showed a grossly fungating mass with focal surface ulceration in the medial side of the foot. The mass was partially necrotic and grossly infiltrated the tendons but did not reach the underlying bone.

**Figure 2:**
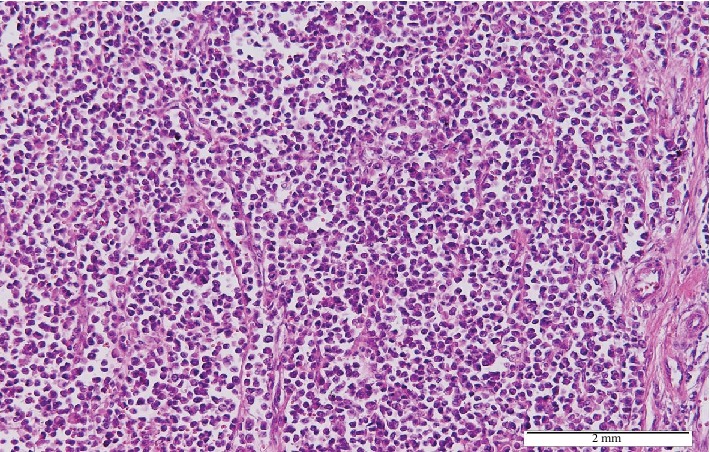
Low-power examination of the tumor with diffuse sheets of round to oval atypical cells separated by thin fibrous septae (H&E × 100).

**Figure 3:**
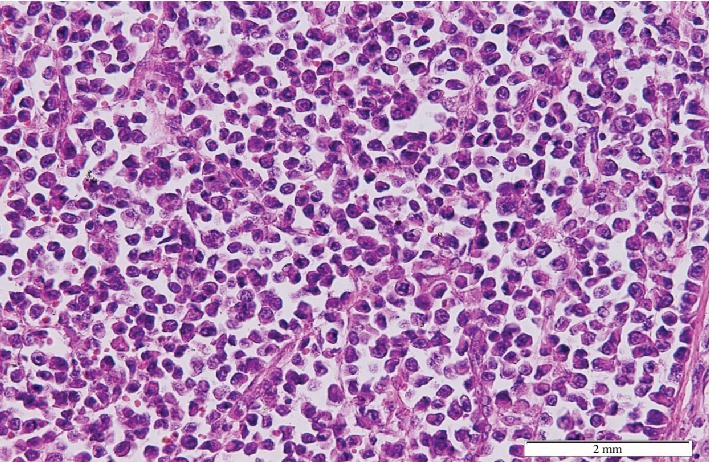
High-power examination reveal pleomorphic nuclei, clear to eosinophilic cytoplasm, and abnormal mitotic figures (H&E × 100).

On microscopic examination of the lesion, a biopsy showed deep dermal infiltration of loosely cohesive, round to oval malignant cells exhibiting eccentric nuclei with a high nuclear to cytoplasmic ratio, abundant clear to eosinophilic cytoplasm, and abnormal mitosis (mitotic count, 25/10 HPF) (Figures 2 and 3).

A panel of immunohistochemical markers was used for testing. The malignant cells were positive for Vimentin, S100, HMB45, MART-1, and CD99 and negative for the remaining markers.

Written consent and the approval of the patient was obtained before writing this report. Ethical approval was also obtained from the Unit of Biomedical Ethics at King Abdulaziz University Hospital.

## 3. Discussion

Clear cell sarcoma was first described in 1965 by Dr. Franz Enzinger [7]. CCS most commonly affects adolescents and young adults [1]. Few reports have shown that CCS predominantly affects females, and recent literature has shown that CCS is equally distributed among both genders [2, 4, 7, 8, 11]. CCS of a primary dermal origin is extremely rare, and it has been reported in remarkably few case studies and series. CCS usually presents as a small mass that involves the foot and ankle. Involvement of the head and neck, kidneys, gastrointestinal tract, and trunk have also been described [1]. In our case, the patient presented with 2 masses, and the largest had an open wound with foul-smelling purulent discharge, which is an unusual presentation for this malignancy and led to the delay in diagnosis in this case.

The diagnosis of CCS is confirmed by histopathological examination of the sample with the help of immunohistochemical staining.

On microscopic examination, CCS usually presents as a fascicular to nested growth of fusiform cells with a diffuse sheet-like pattern of plump polygonal or spindle shape [12, 13]. It also has centrally located ovoid nuclei with abundant clear or pale eosinophilic cytoplasm with low mitotic activity [1, 3, 14]. Immunohistochemical analysis in CCS is positive for HMB45 in 90% of the cases, for microphthalmia transcription factor (MITF) in 71%, for S100 protein in 64%, and for Melan-A markers in 43% [1]. A cytogenic hallmark for CCS diagnosis is a reciprocal translocation, t(11.21)(q13;q12), which results in EWSR1/ATF1 chimeric transcription [3, 4, 14].

MRI is usually used to assess the characteristics of the lesion and the degree of extension. CCS usually presents as a well-defined, symmetrical mass with high-intensity enhancement and strong T1-weighted images in comparison to muscular structures [15]. Other differential diagnoses for masses in the extremities and CCS include clear cell myelomonocytic tumors, malignant peripheral nerve sheath tumors, dermal melanocytic tumors, and synovial sarcoma [1]. Previous literature has shown that CCS has a mortality rate ranging between 39% and 74% in previously described cases [11, 12, 16]. CCS has multiple suggested negative prognostic factors. Previous literature has shown that a large tumor size and the presence of tumor necrosis under microscopic examination are negative prognostic factors in patients with CCS. Other studies have suggested that the extent of the tumor, gender, stage of the malignancy, mitotic index, and surgical margins are prognostic factors [11, 17].

The first-line treatment in the management of CCS is wide local surgical resection of the tumor [18]. However, CCS tends to reoccur locally after treatment. CCS usually metastasizes and involves lymph nodes early. Almost 14% of patients will develop regional lymph node metastasis, and most of these patients will subsequently develop distant metastasis. The most common sites for metastasis after lymph nodes are the lungs and bones [3, 7, 11, 13, 16, 19, 20]. According to a retrospective review and analysis of 31 cases, patients with lung or lymph node involvement have a 0% 10-year survival rate [7, 14, 16]. In our case, the patient had lymph node metastasis, which indicated a poor prognosis. Patients with CCS need to follow up regularly [17, 19]. Some previous studies have recommended annual chest X-rays and PET scans regardless of the length of follow-up because of the high risk of recurrence in the lung after 5 years [14]. After complete excision of the lesion, adjuvant therapy is not recommended [15]. Amputation of the affected limb is not usually recommended unless there is neural or vascular involvement, and multiple studies have shown that both wide local excision and amputation have the same prognosis and survival rate [12, 18]. Chemotherapy is usually reserved for patients with metastatic disease [11, 15, 17, 18].

## 4. Conclusion

The incidence of CCS has not been estimated. CCS should be suspected in any lower limb edema or mass that has developed over a long period of time. Early diagnosis of CCS is necessary to prevent early metastasis, as CCS with metastasis has an extremely poor prognosis. Further studies to assess the incidence, presentation, and management of CCS are recommended to further improve the outcomes in patients with CCS.
